# Fabrication of Weak C-Axis Preferred AlN Thin Film for Temperature Measurement

**DOI:** 10.3390/s21165345

**Published:** 2021-08-08

**Authors:** Ling Dong, Yang Li, Jingwen Lv, Hongchuan Jiang, Wanli Zhang

**Affiliations:** 1 State Key Laboratory of Electronic Thin Coating and Integrated Devices, University of Electronic Science and Technology of China, Chengdu 610054, China; 18589941163@163.com (L.D.); 18328074672@163.com (J.L.); wlzhang@uestc.edu.cn (W.Z.); 2 School of Power and Energy, Northwestern Polytechnical University, Xi’an 710129, China; li2313954@163.com

**Keywords:** weak C-axis preferred AlN thin film, annealing, lattice defect, MATLAB, temperature measurement

## Abstract

A weak C-axis preferred AlN thin film with a lot of defects was fabricated for temperature measurement. It was found that the (002) diffraction peak of the thin film increased monotonously with the increase in annealing temperature and annealing time. This phenomenon is ascribed to the evolution of defects in the lattice of the AlN film. Therefore, the relationship between defects and annealing can be expressed by the offset of (002) diffraction peak, which can be used for temperature measurement. Furthermore, a temperature interpretation algorithm Equation based on the lattice parameter (2*θ*), annealing temperature and annealing time was established, and a temperature interpretation software was built with MATLAB. Visual temperature interpretation is realized by the software, and the relative error is less than 7%. This study is of great significance for promoting the accurate temperature measurement on the surface of high temperature component.

## 1. Introduction

Accurately measuring the surface temperature of the turbine blade and mastering its temperature distribution is an important basis for diagnosing turbine blade breakdown. Surface temperature measurement methods of turbine blades mainly include thin-film thermocouple [1,2,3], temperature-indicating paint [4], infrared radiation [5,6,7], and irradiation crystals [8,9,10]. Thin-film thermocouple technology has the advantages of high integration, but it is not applicable for high-speed rotating blades. Temperature-indicating paint causes no damage to the structure of the test component, but the testing accuracy is very low. Infrared radiation temperature measurement technology is especially suitable for the temperature measurement of high-speed rotating objects, but the variation of emissivity of the tested parts would bring great errors to the test results. Irradiation crystal temperature measurement technology can measure the temperature accuracy by arranging test points in a high density, and it is free from the connecting lead. However, the temperature testing process is quite complicated and expensive. Based on the advantages of irradiation crystals, an easy and cheap temperature measurement method by thin film crystal was proposed. It was reported that the crystal quality can be reinforced after annealing [11,12,13], which indicated that the AlN thin film is a promising candidate for temperature measurement.

This work is dedicated to studying the relationship between the lattice structure and annealing more systematically. We try to establish an algorithm Equation based on the lattice parameter (2*θ*), annealing temperature and annealing time, and develop software to realize the visual temperature interpretation. 

## 2. Experimental Details

The weak C-axis preferred AlN thin film was deposited on alumina ceramic substrate with medium frequency (MF) reaction magnetron sputtering (JGP560) at 300 °C. A high vacuum pressure of 5 × 10^−4^ Pa was obtained by a primary mechanical pump coupled to a condensate pump. A pair of square aluminum targets (200 × 100 × 10 mm, 99.99% purity) and high purity argon and nitrogen (99.99% purity) were used as the sputtering source material for depositing the AlN thin film. Before deposition, alumina ceramic substrate was ultrasonically cleaned in acetone, absolute ethyl alcohol and deionized water for 10 min, respectively. Then the substate was dried by nitrogen gun and fixed at a distance of 80 mm from the Al target. The films were deposited with different nitrogen content (30%, 50%, 70%, 100%, total flow rate of 100 sccm), a working pressure of 0.8 Pa, and MF power of 2000 W. The weak C-axis preferred AlN thin film, with a thickness of 2.5 μm, was grown on alumina ceramic substrate in a growth time of 1.5 h. 

The film deposited with pure nitrogen was annealed in vacuum from 400 to 1000 °C for 40 and 80 min. The pressure of the tube furnace was about 6 × 10^−1^ pa merely by a primary mechanical pump. X-ray diffraction (XRD, Ultima IV, CuKα, 40 KV, 40 mA) was applied to investigate the crystal structure of the film. X-ray photoelectron spectroscopy (XPS, Kratos XSAM 800, Al Kα radiation) was used to measure the chemical composition and bonding state of the film. The temperature interpretation algorithm Equation was established, and the temperature interpretation software was built with MATLAB. 

## 3. Results and Discussion

**Figure 1:**
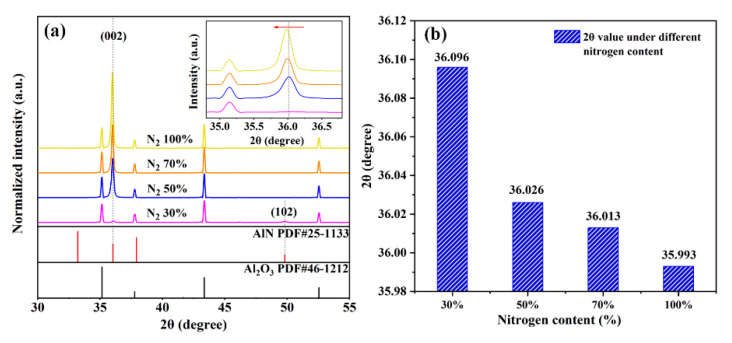
(**a**) XRD pattern of AlN thin films deposited with different contents of nitrogen, the inset is the magnified (002) diffraction peak; (**b**) accurate 2*θ* value of the (002) diffraction peak.

Figure 1a displays the XRD patterns of the AlN thin film deposited with different nitrogen content (30%, 50%, 70% and 100%). Weak C-axis preferred AlN thin film, with a wide full width at half maximum (FWHM), can be prepared with a nitrogen content above 50%, which is consistent with the reported results [14,15]. XRD patterns reveal the characteristic peak of AlN with the hexagonal wurtzite structure. It can be observed that the (002) diffraction peak shifts to a lower angle with the increase in nitrogen content. In particular, the (002) diffraction peak moved by a larger angle when sputtering with pure nitrogen. This result contributes to the greater kinetic energy of nitrogen ion. During the sputtering reaction, nitrogen was ionized into N^5+^ and N^3−^. Compared with Ar^+^, N^5+^ possesses higher kinetic energy due to its high charge. The cations were accelerated to bombard the target, which leads to better crystallinity, but also create more defects inside the film. According to Bragg’s equation, 2*d*sin*θ* = k*λ* [16], where *d* is the interplanar spacing, *θ* is the incident angle, *λ* is the incident ray wavelength, and k is a constant. Figure 1b gives the accurate 2*θ* value of (002) diffraction peak. It can be verified that the interplanar spacing of the (002) crystal plane is expanded by sputtering using pure nitrogen.

**Figure 2:**
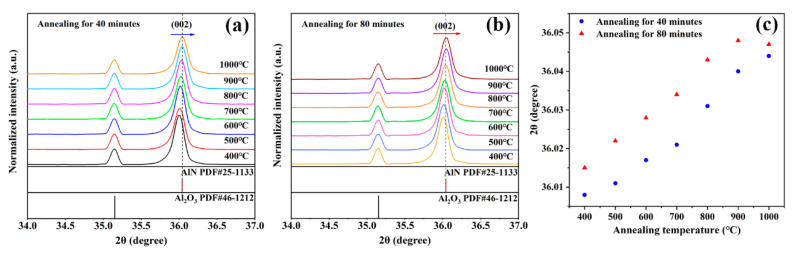
(**a**) XRD patterns of AlN thin films annealing for 40 min; (**b**) XRD patterns of AlN thin films annealing for 80 min; (**c**) 2*θ* values of the (002) diffraction peak for annealed AlN thin film.

Figure 2a shows the XRD pattern of AlN thin films annealed in different temperatures for 40 min. An obvious peak shift towards a higher angle can be observed in the (002) diffraction peak with the increase in annealing temperature. Figure 2b shows the XRD pattern of AlN thin film annealed for 80 min. The (002) diffraction peak also shifts to a higher angle with the increase in annealing temperature. Figure 2c demonstrates that the 2*θ* value of (002) diffraction peak increases linearly with the increase in annealing temperature and annealing time. This result illustrates that the defects were repaired step by step within 1000 °C [17,18,19,20]. However, the (002) diffraction peak returns to a smaller value when annealing at 1000 °C for 80 min, which is due to the fact that some lattice defects were repaired at 1000 °C, but a higher annealing temperature or a longer holding time may generate new lattice defects. Fortunately, the regular linear relationship between the 2*θ* value and annealing (annealed at 900 °C for 40 and 80 min) can be clearly observed. Coincidentally, based on the relationship between the lattice parameter (2*θ*), annealing temperature and annealing time, a novel method for temperature measurement can be put forward.

**Figure 3:**
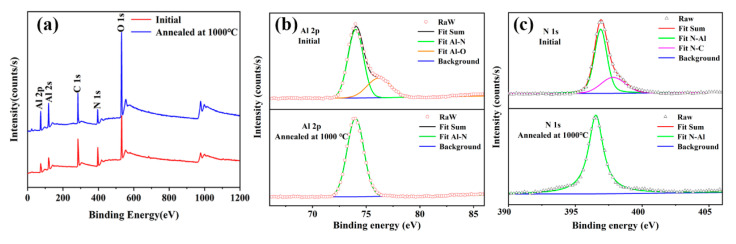
XPS spectra of initial AlN thin film and film annealed at 1000 °C (**a**) XPS full spectra; (**b**) XPS narrow spectra of Al 2p; (**c**) XPS narrow spectra of N 1s.

XPS was carried out to prove that annealing can improve the crystallization quality of the weak C-axis preferred AlN thin film. Figure 3a displays the XPS full spectra of the initial and annealed AlN thin film. The XPS spectra were calibrated by the C 1s peak at 284.8 eV. XPS full spectra confirmed that there is no exceptional element, either in the initial AlN film or the annealed AlN film.

Figure 3b shows XPS fine scans of Al 2p for the AlN thin film. Obviously, a considerable amount of oxygen is observed in the initial AlN thin film, which results from surface-adsorbed oxygen. In contrast, there is no residual oxygen on the surface for the annealed film. That means annealing eliminates impurity atoms on the surface of the film, which leads to a better crystalline thin film [21,22]. The narrow XPS spectrum of N 1s was also laid out to explain the crystalline quality modification of the AlN thin film. As Figure 3c shows, it is obvious that N-C merely exists in the initial AlN thin film. However, an independent Al-N fitting peak is presented for annealed AlN thin film, which illustrates that the surface-contaminated carbon has been removed. This result also represents strong evidence for the suggestion that annealing improves the crystallization quality of the AlN thin film.

The XRD results illustrate that the lattice defects of the weak C-axis preferred AlN thin film can be diminished gradually by annealing, and the movement of (002) diffraction peak directly reflects the relationship between lattice defects and annealing. Consequently, the relationship between annealing temperature, annealing time and 2*θ* values can be established and regarded as the basis of temperature measurement. More specifically, the annealing temperature, annealing time and 2*θ* values are input into MATLAB software to generate matrix sequence. According to the temperature interpretation algorithm Equation reported in the literature [23], we fit the matrix sequence by polynomial in MATLAB software to obtain the temperature interpretation algorithm Equation, as shown in Equation (1).

(1) 2θ=A+B×t+C×T

**Figure 4:**
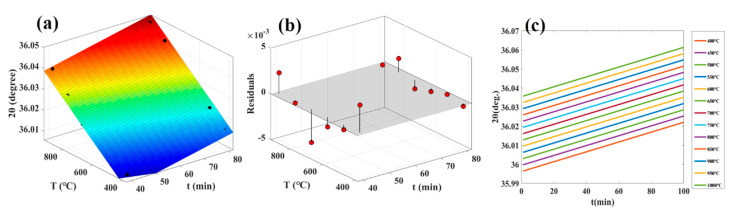
(**a**) The fitted algorithm model diagram of Equation (1); (**b**) the corresponding residual plots; (**c**) standard temperature calibration curve.

In the Equation, *2θ* is the diffraction angle of the (002) plane of the AlN thin film, *t* the is annealing time, and *T* is the annealing temperature. In Equation (1), *A*, *B* and *C* are the constants, the fitting results are 35.97, 0.0002583 and 6.543 × 10^−5^, respectively. The fitting results of different temperature interpretation algorithms are shown in Figure 4, which includes the fitted algorithm model diagram, the corresponding residual plots and the standard temperature calibration curve.

The linear correlation coefficient (R), standard deviation (RMSE) and sum of squared residuals (SSE) of the temperature interpretation algorithms Equations are 0.9912, 0.0019, and 3.2352 × 10^−5^, respectively. The linear correlation coefficient is closer to 1, which means the correlation between variables is stronger [24,25]. Small standard deviation (RMSE) demonstrates that the predicted data are closer to the real data [26,27]. The small sum of squared residuals (SSE) shows that the fitting degree of the linear fitting model is high [28,29]. These data illustrate that Equation (1) is suitable for temperature interpretation algorithm.

**Figure 5:**
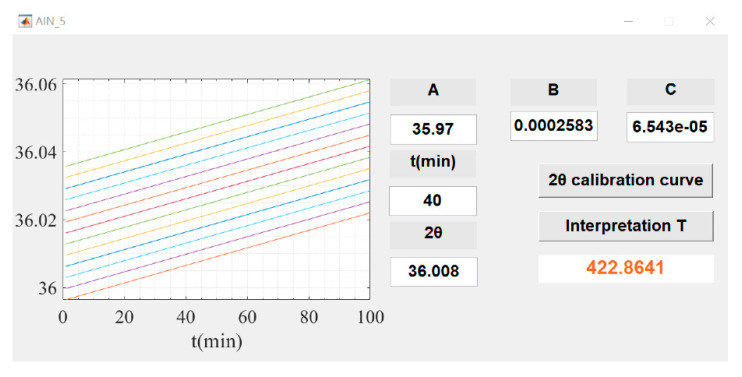
Visual temperature interpretation interface.

In order to realize visual temperature interpretation, temperature interpretation software was built in the MATLAB environment, shown as Figure 5. The temperature interpretation software including axes, text, edit, pushbutton and other objects. For a single object, the corresponding instruction code was written under its callback function. Then, we can input the values of the constants *A*, *B* and *C*, and the temperature calibration curve can be drawn. For temperature interpretation, only the annealing time and lattice parameter 2*θ* needs to be provided, and the maximum temperature experienced by the sample can be read out.

**Figure 6:**
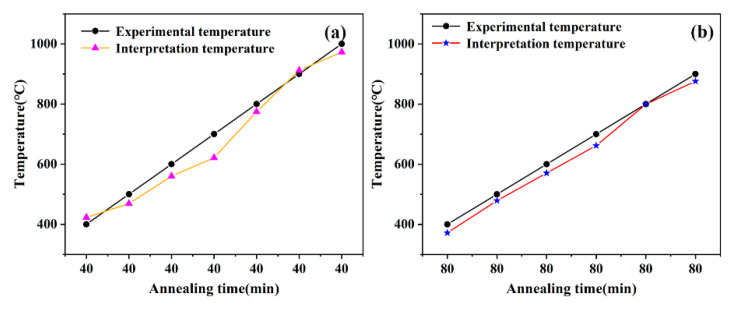
The contrast curve of interpretation temperature and experimental temperature under different annealing time (**a**) 40 min; (**b**) 80 min.

The interpretation temperature is extracted to compare it with the experimental temperature, and the related result is shown in Table 1. As Figure 6 displays, the trend of the interpreted temperature curve is consistent with the experimental temperature curve, but the coincidence degree of the curves is still poor. Therefore, most of the work will be done in the future to improve the accuracy of temperature measurement by the weak C-axis AlN thin film. In this work, except for a few points, the relative error is less than 7%. The relative error is the ratio of the absolute error between the interpreted temperature and the experimental temperature to the experimental temperature. Especially in the higher temperature section, the error of temperature interpretation is smaller, which indicates that the weak C-axis AlN thin film is an ideal candidate for high temperature measurement.

## 4. Conclusions

A weak C-axis preferred AlN thin film with a lot of defects was successfully deposited for temperature measurement. In this work, the relationship between annealing and lattice defects was used for temperature measurement, and the repair of defects by annealing was expressed by the offset of (002) diffraction peak. So, the relationship between the lattice parameter (2*θ*), annealing temperature and annealing time was established to obtain a temperature interpretation algorithm Equation. Moreover, based on the algorithm Equation, the temperature interpretation software was built with MATLAB. Visual temperature interpretation is realized by the software, and the relative error is less than 7%. This study is of great significance for promoting accurate temperature measurement on the surface of high-temperature components. This technology is expected to be applied to the surface temperature measurement of high-temperature components in the aerospace field.

## Figures and Tables

**Table 1:** The data of experimental temperature, interpretation temperature and the relative error.

Experimental T (°C)	Interpretation T (°C)—40 min	Relative Error	Interpretation T (°C)—80 min	Relative Error
400 °C	422.86	5.71%	371.93	7.00%
500 °C	468.71	6.25%	478.92	4.21%
600 °C	560.41	6.59%	570.62	4.89%
700 °C	621.54	11.2%	662.32	5.38%
800 °C	774.38	3.20%	799.87	0.01%
900 °C	911.93	1.32%	876.29	2.63%
1000 °C	973.07	2.69%	\	\

## Data Availability

Not applicable.
